# Two large-scale forest scenario modelling approaches for reporting CO_2_ removal: a comparison for the Romanian forests

**DOI:** 10.1186/s13021-021-00188-1

**Published:** 2021-08-21

**Authors:** Viorel N. B. Blujdea, Richard Sikkema, Ioan Dutca, Gert-Jan Nabuurs

**Affiliations:** 1 Faculty of Silviculture and Forest Engineering, Transilvania University of Brașov, Șirul Ludwig van Beethoven 1, 500123 Brașov, Romania; 2 Forest Ecology and Forest Management Group (FEM), Wageningen University and Research, Droevendaalsesteeg 3a, 6708 PH Wageningen, the Netherlands; 3 Department of Sustainability, Buckinghamshire New University, Queen Alexandra Road, High, Wycombe, HP11 2JZ UK; 4 Wageningen Environmental Research, Wageningen University and Research, Droevendaalsesteeg 3a, 6708 PH Wageningen, The Netherlands; 5 Department of Biological Sciences, University of Limerick, Limerick, V94 T9PX Ireland

**Keywords:** CBM-CFS3, Data harmonization, EFISCEN, Forest sink, NFI, Romania, Forests available for wood supply

## Abstract

**Background:**

Forest carbon models are recognized as suitable tools for the reporting and verification of forest carbon stock and stock change, as well as for evaluating the forest management options to enhance the carbon sink provided by sustainable forestry. However, given their increased complexity and data availability, different models may simulate different estimates. Here, we compare carbon estimates for Romanian forests as simulated by two models (CBM and EFISCEN) that are often used for evaluating the mitigation options given the forest-management choices.

**Results:**

The models, calibrated and parameterized with identical or harmonized data, derived from two successive national forest inventories, produced similar estimates of carbon accumulation in tree biomass. According to CBM simulations of carbon stocks in Romanian forests, by 2060, the merchantable standing stock volume will reach an average of 377 m^3^ ha^−1^, while the carbon stock in tree biomass will reach 76.5 tC ha^−1^. The EFISCEN simulations produced estimates that are about 5% and 10%, respectively, lower. In addition, 10% stronger biomass sink was simulated by CBM, whereby the difference reduced over time, amounting to only 3% toward 2060.

**Conclusions:**

This model comparison provided valuable insights on both the conceptual and modelling algorithms, as well as how the quality of the input data may affect calibration and projections of the stock and stock change in the living biomass pool. In our judgement, both models performed well, providing internally consistent results. Therefore, we underline the importance of the input data quality and the need for further data sampling and model improvements, while the preference for one model or the other should be based on the availability and suitability of the required data, on preferred output variables and ease of use.

**Supplementary Information:**

The online version contains supplementary material available at 10.1186/s13021-021-00188-1.

## Background

Forests play a very important role in the global climate, mainly through their influence on the global carbon cycle [1]. Within the Paris Agreement and subsequent processes, this role of the forestry sector was recognized as an option to mitigate greenhouse gases (GHG) emissions [2, 3] by strengthening the sink function of forests and forest sector. In order to provide confidence in the actual contribution of this sector, reliable monitoring and reporting of carbon flows is essential, and it will become especially important when the Paris Agreement-related global stocktakes will take place in 2023 and 2028 [2, 4]. Namely, if the baseline assessment of the forest carbon balance is not regarded as credible, the mitigation impact of measures will not be accepted either [5–8].

To address the complexity of carbon-flow quantification, several forest models and simulators have been developed since the early 1990s. They range from large-scale empirical forest-stand level decision-support systems such as MELA and Heureka [9, 10], to continental land-use or global land-vegetation models such as GLOBIOM, Orchidee or Lund-Potsdam-Jena model [11–13]. These models simulate the future developments of forest, forest carbon dynamics and also woody biomass availability [14, 15]. However, the models that were often used for policymaking purposes by the European Commission and various European countries are the European Forest Information Scenario Model (EFISCEN), and the Carbon Budget Model of the Canadian Forest Sector (CBM).

The CBM model (version CBM-CFS3 1.2) was originally created to be applied to the Canadian forest inventory, having the aim to perform an inventory of the carbon stock and stock changes in managed and non-managed forests [16]. Nevertheless, CBM was used to simulate even-aged and uneven-aged stands and coppice forests in Europe [17], given the flexibility of user-defined inputs by mixing different shares of growth functions of even-aged single species stands and parameters for simulation of all C pools. The CBM is an inventory-based, yield- and growth-data –driven model for even-aged stands that simulates the carbon dynamics of above- and below-ground biomass, litter, dead wood and soil pools at regional or landscape level [17]. The model identifies five biomass pools (i.e., merchantable, other wood, foliage, fine roots and coarse roots), the transfers from any biomass pool to the wood products pool, as well as C losses in wildfires [16]. Furthermore, the dead organic matter (DOM) consists of 12 sub-pools, which can be aggregated as the three pools—litter, dead wood and soil organic matter—defined by IPCC [18]. Carbon stocks and fluxes to the atmosphere are simulated with a one-year time step, following the United Nations Framework Convention on Climate Change (UNFCCC) reporting requirements [18, 19] for national GHG inventories. During the model run, a library of tables of the standing stock volume and its net increment defines the biomass production by age classes and forest types. Any type of anthropogenic intervention (e.g., thinning, clearcutting, salvage logging) can be applied by CBM, by defining a set of “eligibility criteria” and the specific impact on each C pool [20, 21]. Silvicultural interventions are defined as intensity on standing volume, area or proportion. Their specification can be further defined at more detailed scale, specific to each forest inventory. Moreover, CBM can assimilate dynamically any type of natural disturbance, as well as tree-species change, at any time during the simulation. The model performs a soil-initialization process through iterations until the amount of carbon in slowly decaying DOM pools converges to less than 1% difference at the end of two successive rotations. Once this steady state has been reached by soil-specific pools, the model grows each forest stand to the initial year age class by applying the corresponding yield table (a quasi-equilibrium approach) with the intention to ensure a smoother transition to first simulated years. During the model run, the biomass growth of three above-ground and two below-ground sub-compartments is allocated as a function of merchantable volume. The simulator transfers carbon to and among DOM pools and their emissions to the atmosphere.

EFISCEN (European Forest Information Scenario Model, version EFISCEN 4.2.0) is a detailed forest resource model (wood stocks, increment, harvests) based on about 5000 forest types in Europe, while allowing new data and parameters to be incorporated. It depicts forest areas at regional scale (e.g., NUTS-2) in terms of age classes, growing stocks and increment, using data obtained from the latest available national forest inventory data [22–27]. It has been used to investigate the impacts of forest-management changes, biomass availability and carbon balances [24]. It has also been applied to set the forest management reference level of EU forests under the Kyoto Protocol’s second commitment period [28] and to establish appropriate harvesting levels given the forest-management reference level after 2020 [29]. EFISCEN simulates stem volume and change over time. It is a Markov-chain—type model in which the state of the forest is represented in matrices as an area distribution over age and volume classes [30]. Ageing is simulated as the area transferred to higher age classes, while growth is simulated as the area transferred to higher volume classes. The model simulates stem growth. Stem volume is then scaled up to whole-tree biomass by applying age-dependent biomass expansion factors (BEF) for the other tree biomass compartments as branches, roots and foliage. The model incorporates an earlier version of the Yasso soil model [31]. Litter and dead wood are added from their various sources (stems, branches, foliage, roots) and divided into litter quality classes; these decay products are transferred to five soil pools driven by climate-sensitive functions. There are two ways of initializing soil carbon stocks in EFISCEN. One is to define the stocks for all litter compartments (as total carbon in the forest type); the other is to run a spin-up in which the litter input of the first time-step is used as input to Yasso, and then Yasso is run repeatedly until the soil stocks converge within 1% difference. The spin-up will run automatically if the initial stocks are set to zero. For the comparison, we used the second method, i.e., to run a spin-up, due to the lack of available data on litter compartments. The factor driving forest management in the EFISCEN model is the harvest regime. Harvest regimes are specified at two levels in the model. First, a basic management regime per forest type and country defines the age range during which thinnings can take place and a minimum age for final fellings. These regimes can be regarded as constraints on the total harvest level. Multiplying the area available for thinnings and final fellings by the corresponding wood harvest gives the volume of wood that is theoretically available for harvesting. In the second step, the actual demand for wood is specified for thinnings and for final felling separately at the national level. Thinning intensity is simulated by transferring area to a lower volume class, while the difference in volume is assumed to be the volume that has been removed by the thinning. Final felling is simulated by moving the area back to the first volume and age class of the matrix, from where it can start growing again. The difference in volume is assumed to be the volume removed by the final cut [27]. EFISCEN can deal with natural disturbances [32], and it allows changes of tree species after a clear cut.

The CBM model has been applied to the EU member states, in order to estimate the contemporary EU forest carbon dynamics from 2000 to 2030 [17, 33, 34]. Other countries used CBM for scientific explorations or operational purposes such as UNFCCC reporting [35, 36]. EFISCEN has also been extensively used in the EU for national forest resource projections, as for example in the forest sector outlook study [37], the woody biomass availability studies [38] and carbon-sink projections under the land use, land-use change and forestry (LULUCF) regulation [29].

Prior studies assessed and improved the reliability of EFISCEN. For example, projections over long periods were performed using historical forest inventory data from Finland and Switzerland. After an additional uncertainty analysis for both countries, the EFISCEN model was refined [39, 40] in terms of parameterization of growth functions and in terms of management regimes. EFISCEN was also subject to quality assessment and sensitivity analysis, being made available as an open access software [26]. The CBM model has also been extensively investigated. For example, an uncertainty assessment was first executed for the dead organic matter pool in managed forests [41]; then, the accuracy of CBM was investigated by comparison with independent National Forest Inventory (NFI) estimates [42]. Metsaranta et al. [43] explored the accuracy of CBM estimates by using Monte Carlo simulation approaches to propagate errors from model parameters and other variables in order to obtain confidence intervals for carbon stocks and fluxes. Besides these accuracy-oriented studies, additional research has been focused on improving the decomposition module [44]. However, in a quantitative analysis of the land-trade–industry framework [45], the maximum wood supply in the EU was estimated using CBM and compared with that obtained earlier (with different input data) by using EFISCEN [46]. On average, CBM estimates of potential woody biomass supply were 20% larger than EFISCEN estimates, for which the reasons stayed unclear. Therefore, a deeper understanding of the causes of these differences is necessary.

As one way of model validation, we assessed the reproducibility of CBM (CBM-CFS3 version 1.2) and EFISCEN (version 4.2) by comparing the results of simulations using harmonized assumptions and inputs derived from the same underlying data. To initialize both models, the data of the NFI (NFI-1 and NFI-2 [47]) of Romania was obtained. This represents the state of the forest around 2010, while our study covered the forest available for wood supply. Thus, the aim of this study was to analyse the harmonization challenges and to assess the maximum level of agreement of CBM and EFISCEN projections at the national level for the forest carbon pools. We performed a quantitative comparison of forest indicators, carbon stocks and CO_2_ fluxes as simulated for Romanian forests available for wood supply, up to 2060. We identify and explain differences originating from the two modelling approaches. We focused mainly on processes affecting living biomass (i.e., standing stock, growth and mortality) and less so on forest soils (although they are also reported in the paper). Romanian forests were selected for this study as they exhibit a wide range of management regimes while their structure is relatively uniform (approximately 85% of these forests are even-aged or relatively even-aged); thus, Romanian forests are suitable for these models and are reasonably characteristic for European forests, without a lot of bias on either boreal or Mediterranean characteristics.

## Results

### Dynamics of the forest indicators

**Fig. 1:**
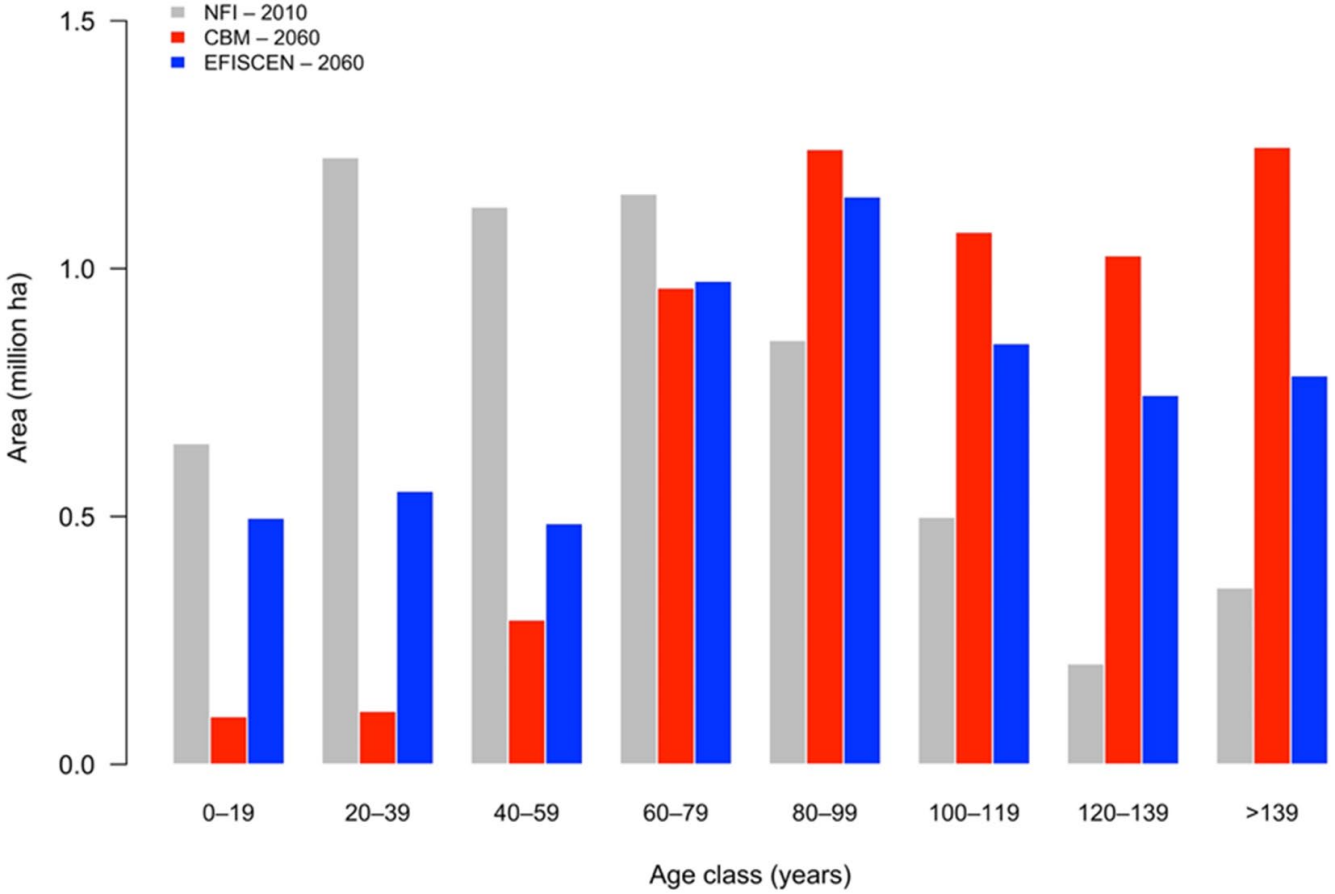
The dynamics of age class for 2010 as assessed in the NFI and as simulated by CBM and EFISCEN for 2060. The default outputs of 10-year age classes in EFISCEN were aggregated to match the default output of 20-year age classes in CBM

In Fig. 1, the CBM and EFISCEN results for the forest area distribution by age class at the end of simulation period are compared with the NFI assessment at the beginning of simulation period. Both models showed an increase of forest area in older age classes by 2060. However, CBM shows a stronger ageing effect, although the same volumes of wood were demanded for national harvest in both models. EFISCEN simulation leads to a more even distribution over age classes with more regeneration. CBM simulated a 55% larger area in the oldest age class (above 139 years).

**Fig. 2:**
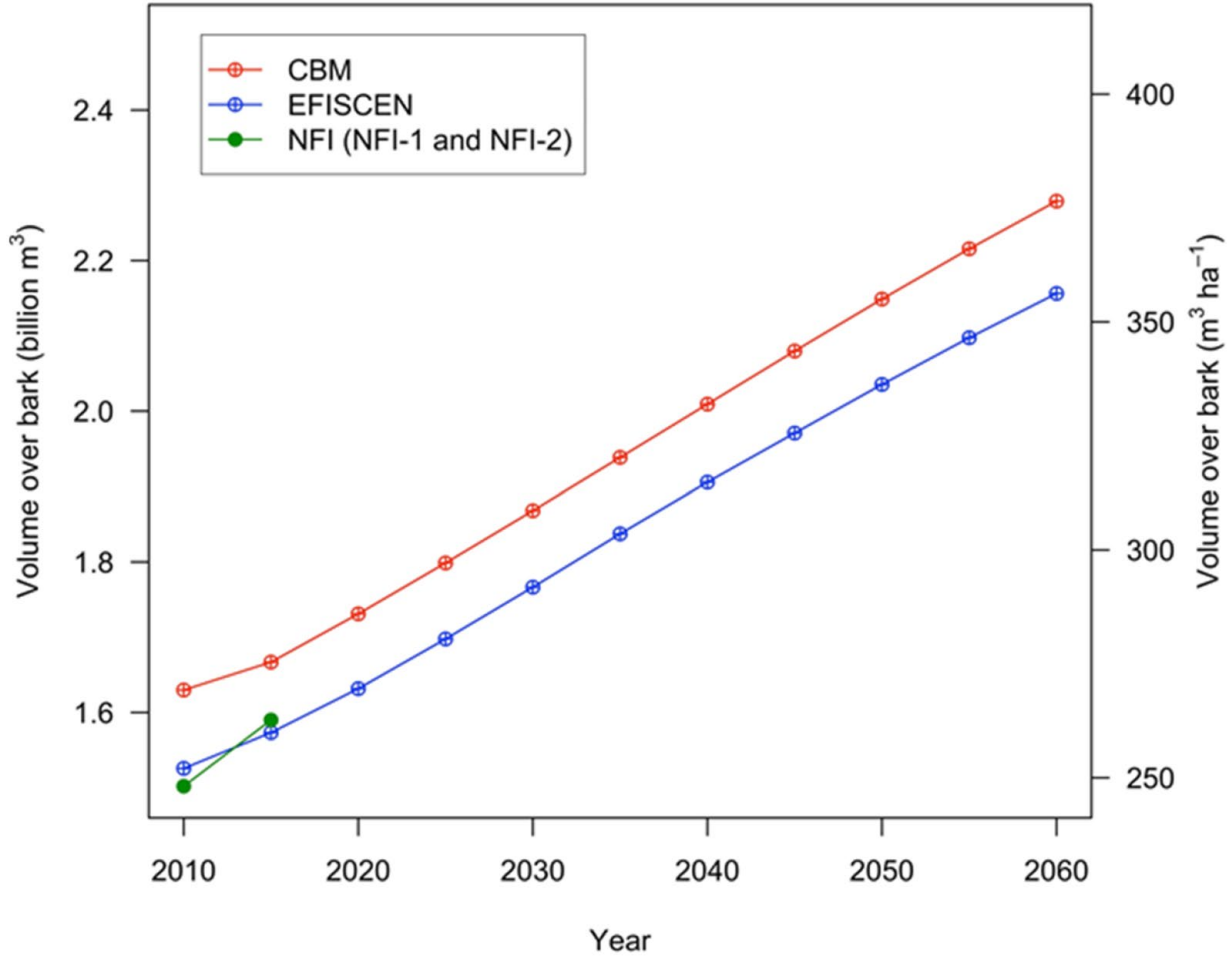
The merchantable standing stock (standing volume over-bark) development over time, simulated by CBM and EFISCEN for FAWS

In 2010, the forests available for wood supply (FAWS) area of the NFI-1 consisted of 17% coniferous, 63% broadleaved and 20% mixed forests (NFI-1). By 2060, the total simulated area decreased negligibly because of deforestation, which was distributed randomly across age classes and forest types by CBM, and extracted from area subject to final cuts by EFISCEN. In Fig. 2, the simulated development of the merchantable standing stock (see definition in Additional file 1: Appendix S1) is provided as well as its comparison to the stocks provided by the two NFIs. EFISCEN started close (+ 1.5%) to the initial data from the NFI-1 (247 m^3^ ha^−1^) and matched the data for the NFI-2 well; CBM initialized a merchantable standing stock 6.6% larger than the NFI-1 estimate. Disaggregating the initialized merchantable standing stock by forest type, CBM presented 18% and 12% smaller standing stocks for coniferous and mixed forests, respectively, and 15% larger standing stock for broadleaves, compared with EFISCEN and NFI estimates.

At the end of the simulation period, EFISCEN reached 358 m^3^ ha^−1^, whereas CBM topped at 377 m^3^ ha^−1^. Overall, the slope of the merchantable standing stock increase was very much the same between the models. The merchantable standing stock differed by 6.8% in 2010 and by 8.1% in 2060.

Because of slight differences in harvesting regimes, the merchantable standing stock over time per species group differed slightly between the models. In EFISCEN, the proportion of coniferous forests in the merchantable standing stock increased from 32 to 33% and the broadleaves decreased from 68 to 67% during the simulation. In CBM, the proportion of broadleaves increased from 55 to 59% and remained at 25% for mixed forests, whereas for coniferous forests, it decreased from 20 to 16%.

**Fig. 3:**
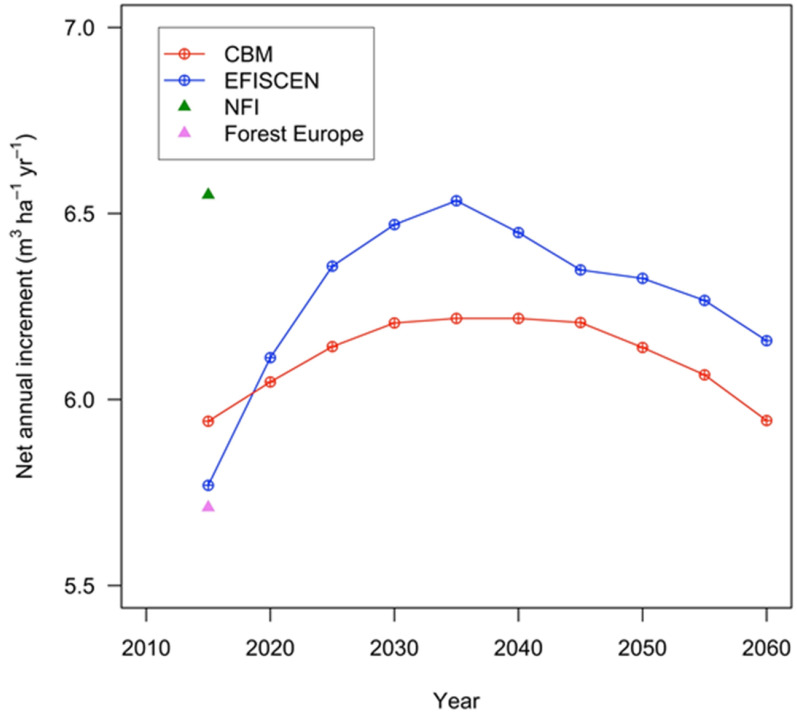
The dynamics of the average NAI over time in the simulation by the two models as well as initial values provided by the NFI and by Forest Europe [48]

CBM projected a 5% lower net annual increment (NAI) on average over the simulated time (Fig. 3). The fairly fast increasing trend of EFISCEN was caused by the development of age class distribution (more regeneration area, see Fig. 1) as well as an enhanced growth after thinning interventions (Additional file 1: Appendix S2). A similar pattern (i.e., an increasing NAI for the first two decades) occurred also in CBM, although CBM did not implement such a thinning response, and it had an older forest developing.

Both models estimated a smaller NAI compared with the NFI, given different approaches to calculate the merchantable standing stock and NAI by the NFI, such as (i) minimum disaggregation at tree-by-tree in NFI compared with stand level in our study and (ii) the simplification of forest complexity to only forest types in the models compared with the NFI.

**Fig. 4:**
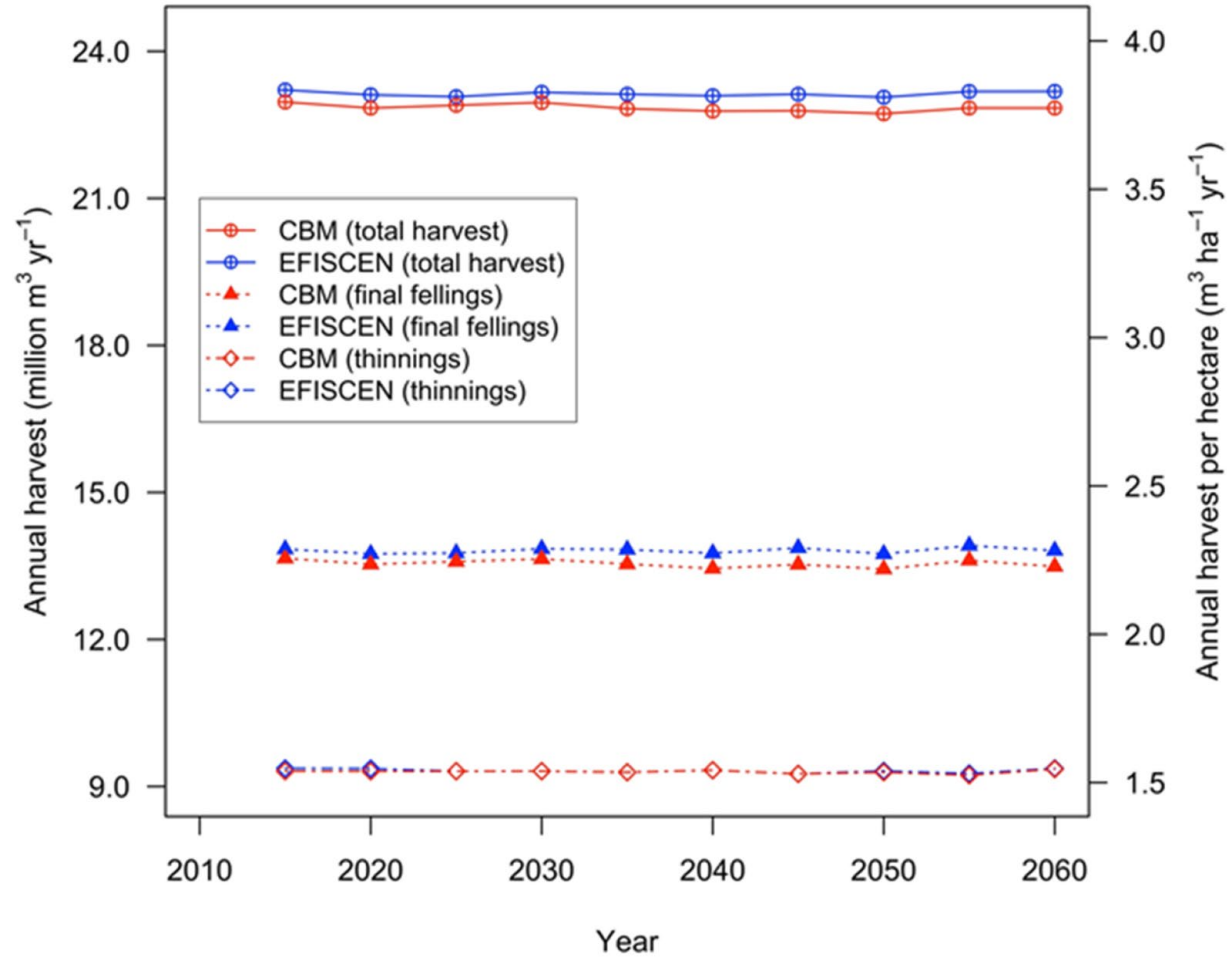
The dynamics of annual thinnings and final fellings, and thus total harvest, as simulated by CBM and EFISCEN for FAWS

Figure 4 shows that the stable demand for harvest, which was a requirement of the modelled forests, was also found at a constant felling indicator of approximately 3.77 and 3.83 m^3^ ha^−1^ yr^−1^ for CBM and EFISCEN, respectively. Both models satisfied a roundwood demand of about 23 million m^3^ yr^−1^ during the simulation period. The total demand to be extracted by thinning was fully satisfied by both models. A minimal difference was found in the final fellings of 2.2–3.3% less in CBM. Throughout the simulated period, CBM underperforms by some 2% the target defined for the final cut. Overall, the total harvested volume consisted of 26% coniferous-, 27% mixed- and 47% broadleaved-based forests.

**Fig. 5:**
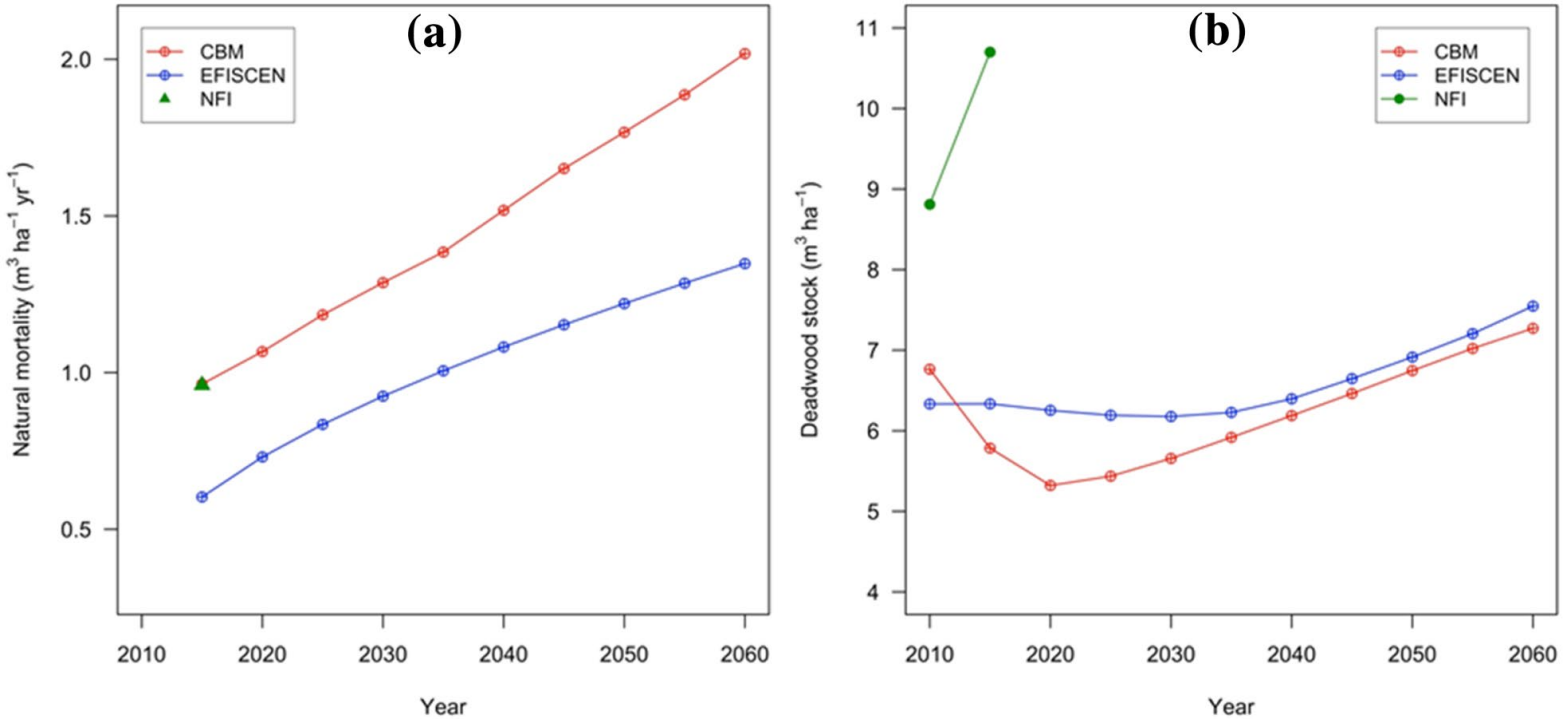
The dynamics of simulated natural mortality over time (**a**) and the dead wood stock (**b**). The higher natural mortality rate in CBM did not result in a higher dead wood stock, because of the higher decay rate

The NFI data revealed a high mortality rate in Romanian forests of 0.96 m^3^ ha^−1^ yr^−1^; see Additional file 1: Appendix S1. The mortality rate is expected to have a significant effect on the standing stock dynamics, as it represents approximately 15% out of the net annual increment. Figure 5a shows the dynamics of natural mortality rate as simulated by the two models. Because CBM initiated a larger merchantable standing stock (Fig. 2) and ended with a larger area of older age classes in 2060 (Fig. 1), this ageing will increase the mortality in CBM. On average, the mortality simulated by CBM was both larger and more accelerated compared with EFISCEN.

The simulated standing dead wood volumes in CBM and EFISCEN in 2010 were, respectively, 25% and 28% less than the NFI-1 (Fig. 5b). For both models, the standing dead wood stock decreased slightly during the first 1–2 decades of the simulation period, followed by an increasing trend that was sustained by the end of simulation period. This pattern arose because, in the first part of the simulation period, the limited mortality was smaller than the decay. However, towards the end of the simulation period, this ratio has reversed (i.e., the mortality started to overtake the decay).

### Carbon stocks and fluxes

**Fig. 6:**
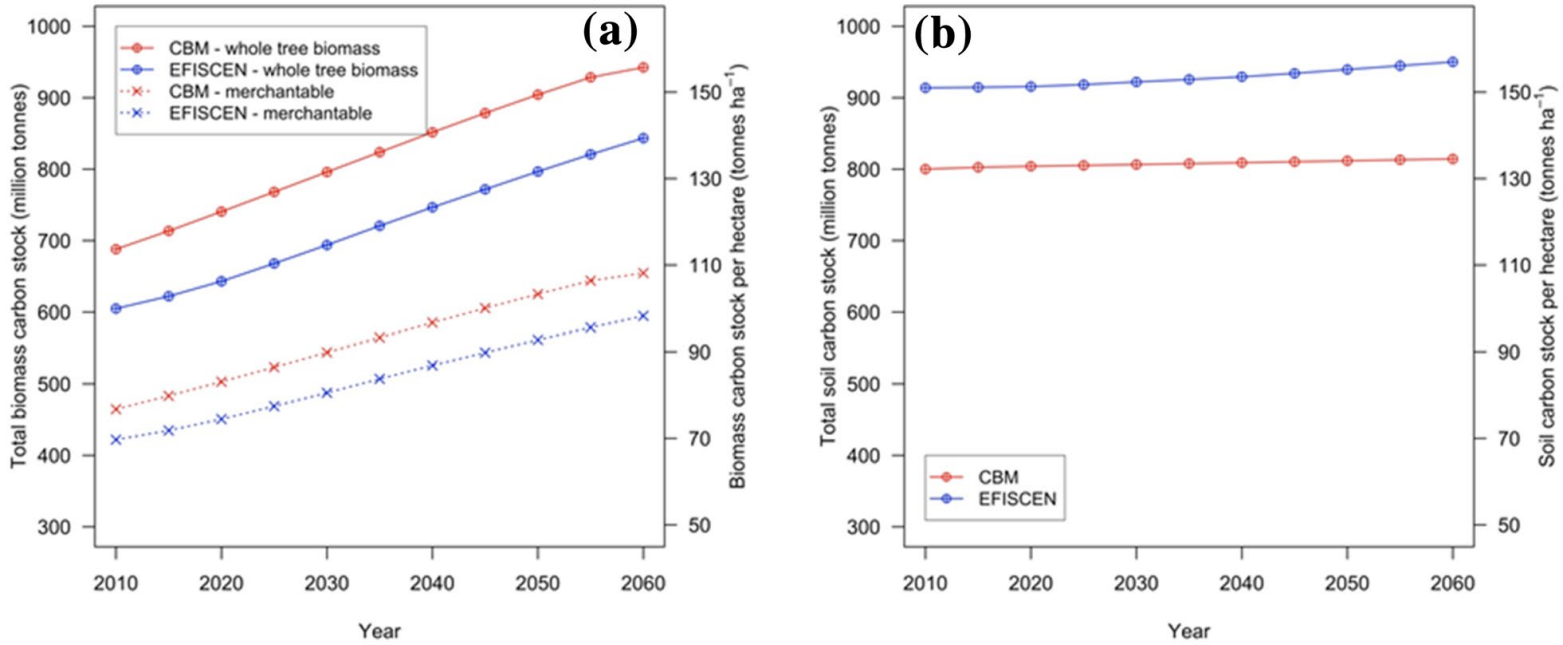
The trends in biomass carbon stock (**a**) and soil carbon stock (**b**)

The carbon in the merchantable standing stock increased steadily in both models over the simulation period. However, systematic differences between models occurred (Fig. 6a). For the initial simulation year (i.e., 2010), there was a 9.9% difference between models and the NFI. In EFISCEN, the total carbon stock was 422 million tC (tonnes of carbon), which is equivalent to 69.6 tC ha^−1^; the corresponding total amount in CBM was 464 million tC (or 76.5 tC ha^−1^). This larger amount simulated by CBM is consistent with the generally higher standing stock volume as well as with the higher share of broadleaves in 2010 initialized by CBM.

The relative difference is maintained fairly constant throughout the simulation period, until 2060, when the total carbon stock reached 595 million tC (98.6 tC ha^−1^) in EFISCEN and 655 million tC (108.3 tC ha^−1^) in CBM. CBM showed an increase of the carbon stock by 41.6%, compared with a 40% increase in EFISCEN. Overall, for 2060, CBM simulated 13% larger carbon stock for coniferous forests (i.e., species with lower wood density), 50% larger carbon stock for broadleaves (i.e., species with denser wood) and 40% larger carbon stock for mixed forests.

During the simulation period, the carbon stock of the total living biomass increased from 113.3 to 155.9 tC ha^−1^ in CBM and from 99.8 to 139.8 tC ha^−1^ in EFISCEN (Fig. 6a). The differences between models decreased from 13.7% in 2010 to 11.7% in 2060. This dynamic is consistent with the larger frequency of old stands in CBM compared with EFISCEN (see Fig. 1), as well as with the wood removals implemented by the two models (Fig. 5).

Despite the harmonization efforts, the allocation in other tree biomass compartments was simulated slightly different by the two models (Table 1). The most substantial difference in merchantable wood (i.e., the largest contributor to total standing living biomass) was caused by the inclusion of tree-tops in EFISCEN. EFISCEN allocated marginally more biomass in the below-ground pool, due to the inclusion of the non-harvestable part of the above-ground stump biomass to coarse roots; CBM integrated that into above-ground biomass instead. CBM simulated a smaller share of foliage biomass compared with EFISCEN.

**Table 1:** The proportions of carbon stock in various biomass compartments, in 2010 and 2060

Model	Simulation year	Merchantable stem^a^	Foliage	Other woody compartments^b^	Coarse roots^c^	Fine roots
CBM	2010	66%	2%	14%	16%	2%
	2060	68%	1%	15%	15%	2%
EFISCEN	2010	70%	3%	9%	16%	2%
	2060	71%	2%	9%	16%	2%

The shares of various components in total carbon stock in the living biomass pool (aggregated for all forest types) estimated based on carbon outputs from the models (percent of total tree biomass):

^a^The “merchantable stem” category was defined in CBM as the stemwood over-bark corresponding to gross merchantable standing wood; in EFISCEN, it was defined as merchantable stem over-bark including tops

^b^The “other woody compartments” category is composed of living branches, tree-tops and above-ground stump (with bark, in CBM). In EFISCEN, the above-ground stumps are included in the coarse roots

^c^In EFISCEN, the “coarse roots” includes the above-ground stump

The total carbon stock in the aggregated DOM pool (i.e., containing dead wood, litter and soil organic matter) was on average 13% larger in EFISCEN than in CBM. Under the specifically defined initialization procedure, the EFISCEN started the simulation from an initialized stock of approximately 900 million tC, whereas CBM started with just under 800 million tC (Fig. 6b). For the simulation period, the average soil carbon stock increased from 151 to 157 tC ha^−1^ in EFISCEN, and from 132 tC ha^−1^ to only 135 tC ha^−1^ in CBM (Fig. 6b). By comparison, in an in-depth study on carbon stock in Romanian mineral forest soils, Dincǎ et al. [49] reported an average soil carbon stock of 137 tC ha^−1^. An in-depth analysis of soil stocks and change on forest types and climates by soils module in CBM and the newer Yasso15 version has been recently reported [50].

**Fig. 7:**
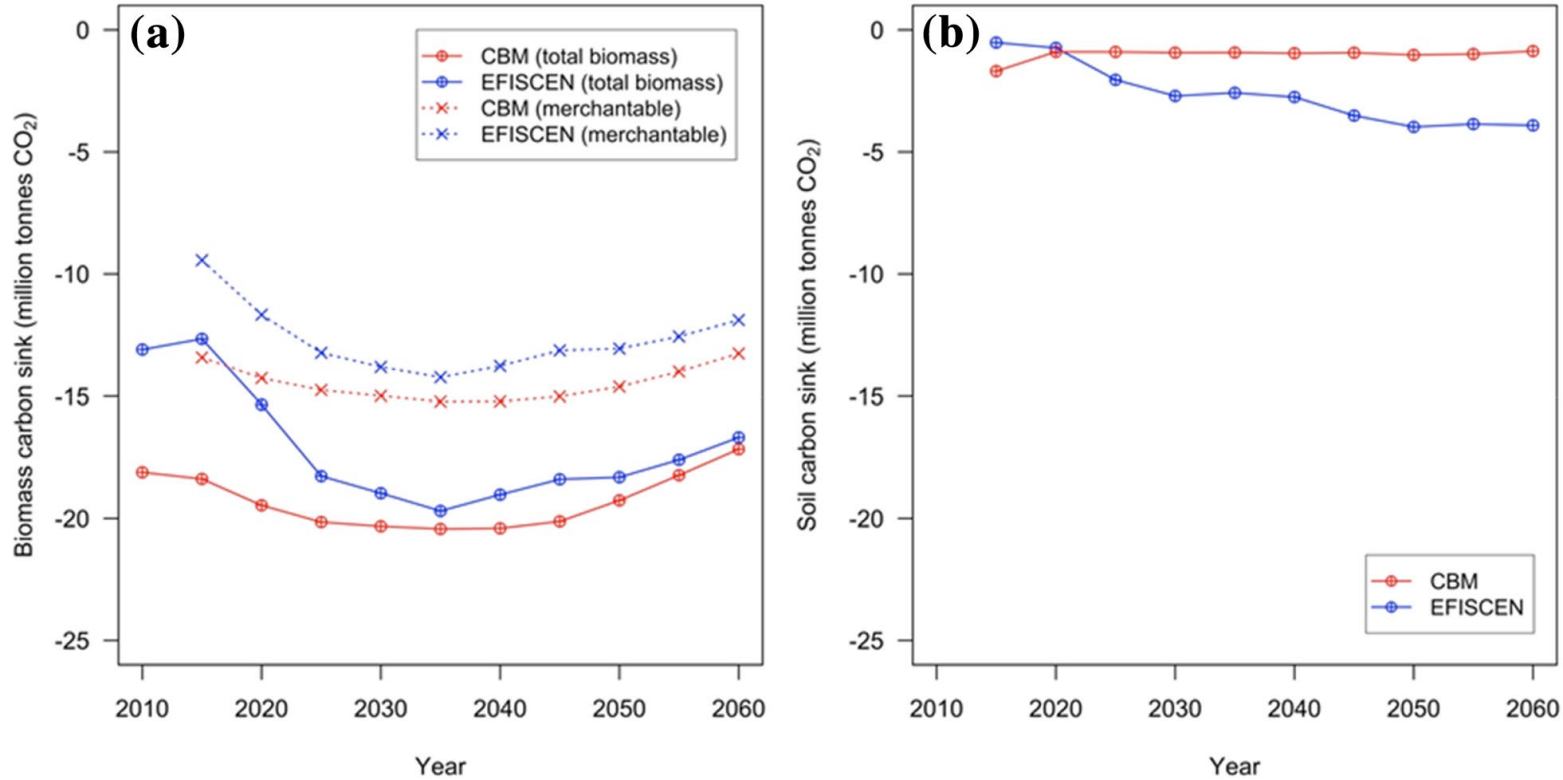
The carbon sinks as simulated by the two models in living biomass (**a**) and soils (**b**), plotted at same Y-axis scales. Negative numbers are sinks

The carbon sink in forest biomass showed an increasing trend for the first half of the simulation period for both models, followed by a descending trend. In EFISCEN, the carbon sink in merchantable stock started from –9.5 million tonnes of CO_2_ (tCO_2_) yr^−1^ in 2015, peaked at –14.2 million tCO_2_ yr^−1^ in 2035 and decreased to –11.9 million tCO_2_ yr^−1^ in 2060. The corresponding values in CBM showed a smaller variation, from –13.2 million tCO_2_ yr^−1^ in 2010, to –15.2 million tCO_2_ yr^−1^ in 2035 and back to –13.3 million tCO_2_ yr^−1^ in 2060 (Fig. 7a). A similar pattern was repeated for the carbon sink in total standing living biomass. In EFISCEN, the minimum sink value occurred in 2015 (–12.7 million tCO_2_ yr^−1^), and in 2035 there was a maximum (–19.7 million tCO_2_ yr^−1^), which decreased to –16.7 million tCO_2_ yr^−1^ in 2060. In CBM, the simulation started from –18.1 million tCO_2_ yr^−1^ in 2010, increased to –20.4 million tCO_2_ yr^−1^ in 2035 and decreased to –17.2 million tCO_2_ yr^−1^ in 2060 (Fig. 7a). Overall, the mean difference between models with regard to carbon sink of the total living biomass was approximately 13%. The difference was largest for the first decade of the simulation period.

This discrepancy occurred through the cumulative effect of various differences between models, as follows: the simulation of slightly different NAI values (see Fig. 3), the proportion of non-merchantable biomass compartments (see Table 1), the impact of different concepts to allocate the harvest demand across forest types (see Fig. 4) and different mortality rates (Fig. 5a). Further, a significant contribution to the difference lies in the increasing share of broadleaved species in the standing volume by CBM, while the felling demand was satisfied by all forest types during the simulation by both models. Overall, CBM simulates an increasing proportion of broadleaved forests (from 55 to 59%) and a decreasing proportion of coniferous forests (from 20 to 16%).

The change in mineral soil carbon was simulated as sink by both models. In CBM, the carbon sink in mineral soil was − 1.7 million tCO_2_ yr^−1^ in 2015, and starting with 2020, it stabilized at around − 1.0 million tCO_2_ yr^−1^. A different trend occurred in EFISCEN. The carbon sink in mineral soil was a minimum in 2015 (− 0.52 million tCO_2_ yr^−1^), increased steadily to − 4.0 million tCO_2_ yr^−1^ in 2050 and stabilized at − 3.9 million tCO_2_ yr^−1^ for the rest of the simulation period, corresponding to larger standing stocks and thus larger litterfall rates. There are explanations for the opposing sink trends. First, the different initialized values may be linked to the quasi-equilibrium procedure in CBM compared with equilibrium one in EFISCEN. CBM’s quasi-equilibrium consists of adjusting initialized C stock with the inputs corresponding to the stands’ age, while EFISCN starts with an equilibrium between litter inputs and soil C, thus always starting from a balanced state.

## Discussion

Comparing models can be seen as an alternative way to validate the credibility of model outcomes and results. These two models belong to empirical forest-inventory–based bookkeeping models. We showed that when maximum harmonization is strived for in the input and scenarios, these models produce comparable results and, to a large extent, the same trends. However, when the models employ different approaches (such as for the soil compartment), the output dynamics differ. Our results highlighted those small differences in modelling principles of CBM and EFISCEN combined with the available data can yield differences in output estimates on various components. Despite the efforts invested in harmonization of the input parameters, differences remained between the estimates of the two models. Here, we discuss in detail the potential causes of these differences.

### Uncertainty of input data

Developing an uncertainty or sensitivity analysis for CBM and EFISCEN was beyond the aim of our study. However, it is obvious that the estimates of both models are affected by uncertainty. According to the NFI [47, 51], the sampling error estimated for each national-scale valid indicator was typically low (e.g., 6% for fellings and 1.7% for the merchantable standing stock). Since the input data were disaggregated, it is expected that the standard error will increase (e.g., up to 12% for highly represented, e.g., *Fagus sylvatica*, Norway spruce and other broadleaved forests, and up to 100% for low represented, e.g., other coniferous and silver fir forests).

For CBM, an important source of uncertainty originates in the fit of yield, increment and Boudewyn models. These models were fitted on nationally available data. For the yield models, the residual standard error of the yield models, also relative to the mean predicted value, varied by forest type between 49 m^3^ ha^−1^, or^−^ 20.8%, for *F. sylvatica* and 172 m^3^ ha^−1^, or 94.8%, for *Abies alba* forest type. For NAI models, the residual standard error varied between 0.68 m^3^ ha^−1^ yr^−1^ for *Robinia pseudoacacia* and 3.2 m^3^ ha^−1^ yr^−1^ for the “Other Broadleaved” forest type. The Boudewyn models, used to predict the proportion of biomass compartments, showed residual standard errors between 1.1% for the bark of “PredBroad” forest type and 11.5% for the proportion of *R. pseudoacacia* stemwood. EFISCEN uses BEF, developed for each forest type and each biomass compartment, by age class. The standard deviation of BEF varied between 6.3% for stem biomass of *A. alba* trees and 0.2% for branch biomass of the “Other Broadleaved” forest type. These standard deviations include, however, the variation by age class. Therefore, the standard deviation of allocation in biomass compartments within age class is expected to be smaller than the range presented.

To reduce the uncertainty sourced in these models (which act as input data in either EFISCEN or CBM), additional observations at tree and stand level should be collected from Romanian forests based on a robust sampling design. Nevertheless, data analysis should engage appropriate model selection criteria to minimize the uncertainty related to model selection.

### Merchantable standing stock

Compared with the NFI estimate, in 2010, the simulated merchantable stock volume was 7% larger in CBM and only 1.5% larger in EFISCEN (see Fig. 2). However, for C stock, the difference between models and the NFI was 9.9%. Furthermore, between the models, the differences in merchantable standing stock volume increased in time (from 6.8% to 8.1%), whereas the differences in C stock reduced (from 13.7% to 11.7%), mainly because of the change in the share among forest types with different wood densities. These differences between merchantable standing stock volume and carbon stock may generate confusion when making climate decisions based on forestry indicators. For example, the forest can be assumed sustainable from a forestry perspective (when the harvest volume is slightly lower than the increment volume); however, it may be assumed not sustainable from a corresponding climatic perspective (C from harvest is larger than C from forest growth). This may happen, for example, when harvesting more broadleaved species with higher wood density (therefore the unit of volume contains more biomass and more carbon), while the growth of living biomass is ensured mainly by coniferous species (with generally low wood density).

These differences originated in the way CBM reconstructed initial standing volume from user-defined yield tables, while EFISCEN used the exact values reported by the NFI. On forest types, the actual deviations of the initial standing stock were up to ± 20% for the values initialized by CBM. We identified two limitations that affected the fit of merchantable standing stock volume models: (i) eight out of 10 forests types were subject to shelterwood systems, therefore, with lower standing stocks for stands older than 100 years, for which reason the developed yield curves predicted systematically larger stock (assuming that standing stock was not reduced by the shelterwood system); (ii) the yield curves were derived from age class–dependent standing stock volumes per forest type and per owner type, available as region averages (at NUTS-2 level) as no detailed information at the NFI plot level was available. The NFI estimate resulted from much more detailed stratification on approximately 25 forest types [47, 51] compared with our structure of 10 forest types. A further simplification has to do with omitting the specific models for mixed forests, despite the existing knowledge on the growth of mixed tree species [52]. Moreover, we did not include in the simulation the effect of specific regional [53] or local growth conditions as the site index. This omission may further affect to some extent the accuracy of growth and yield projections in both models at the more local scale. In fact, both models can deal with growth functions that would represent the share of each species in a mixed forest, but that is not seen as a mixed-forest model.

The C stocks reported here are within the typical range of standing C stock for most of the European forests [48]. Comparatively, Bouriaud at el. [54] found that above-ground biomass in Romanian beech forests increased with stand age across all management types and treatments, reaching about 300 t dry biomass ha^−1^ equivalent to approximately 150 tC ha^−1^ at the age of 100 years, similar to our estimate. Similar estimates of standing stock were reported by the National Forestry Accounting Plan of Romania [55], where the yield table was based on models developed by Giurgiu and Draghiciu [56]. We question whether the initial overestimation of the merchantable stock has any marginal impact on the accuracy of the CBM-based estimates. We speculate that the overestimated initialized standing stocks propagate until the end of the ongoing production cycle, while applying an inaccurate increment would affect both the first and the following cycles.

Furthermore, we could not detect any substantial effect of CBM annualization of the area in the initial simulation year (i.e., CBM divides the input area for each age class in 10 equal areas corresponding to a one-year time step).

To keep the required initialization data to a minimum, only the area and the mean growing stock volume per age class as reported by the NFI were retained in EFISCEN for the initial year of simulation. For the simulation period, the volume distribution over age classes (matrix columns) was generated by an empirically based function [26]. The aggregation of all individual volumes to a nationally aggregated volume in a different way than the NFI may have caused the 1.5% overestimation in EFISCEN (compared with the NFI estimate).

Through the Results and Discussion sections, all elements related to merchantable stock in the initial year of the simulation pertain as validation effort. Using an additional evaluation, we assessed that the yield curves used in CBM practically matched the Romanian yield curves corresponding to site productivity between the third and fourth class [56].

### Net annual increment

Because of the particular type of indicators in Romanian forestry addressing the volume of the entire above-ground woody biomass, it is practically impossible to validate NAI of merchantable part only as used in this modelling exercise, without making assumptions.

Despite differences regarding the initial merchantable standing stock, its relative rate of change was simulated similarly by both models, although EFISCEN showed a slightly higher increment rate. This may be related to the enhanced regeneration that takes place in EFISCEN leading to a younger age class distribution. Also, less area harvested by CBM, given higher merchantable standing stock, leads to overall older forests. It is worth noting that EFISCEN implements the volume-increment dynamics as a percentage of the growing stock [26]. On the other hand, CBM requires user-defined volume-increment functions, so the accuracy as well as the consistency of yield and increment curves depend on the user-selected pre-processing operations, i.e., independent of the modelling frame of the CBM model.

### Harvesting algorithms

The harvesting allocation algorithm had a noticeable effect on the estimates of all forest indicators because it affects age classes, thus also increment, thus also the sink, etc. Both models satisfied a demand of 23 million m^3^ yr^−1^. Harvest allocation also had an effect on merchantable stocks per species. In CBM, a low harvest rate of broadleaved forests, according to historical rate, resulted in the increasing proportion of broadleaves and the decreased proportion of coniferous forests in standing stock. After 50 simulated years of forest management, the standing stock was composed of relatively more broadleaved forests (i.e., of species with higher wood density) and fewer coniferous forests, contrary to what is simulated by EFISCEN. This also explains the higher C stock in 2060 in CBM compared with EFISCEN.

Conceptually, the implementation of harvest is very different in CBM compared with EFISCEN. EFISCEN applies a so called “free allocation”, while CBM uses a “detailed allocation”. The free-allocation approach allows EFISCEN to manage internally the split of total harvest demand on thinning and final fellings by species based on pre-defined management regimes per species and age class. The EFISCEN model then searches for its total requested demand, given the state of the forest, and allocates it across available forests types. In CBM, for an optimum projection of the forest-management interventions, the harvest needs to be projected by an external effort, e.g., allocation of harvest based on simulated future dynamics of standing stock, as in ref. [33], or through specific tools, e.g., the Remsoft Spatial Planning System as used in Canada [57]. Most likely, the constant harvest scenario that we applied was satisfied given the relatively low share of the harvest rate from the total increment rate (some 66%) or from available standing stock subject to applicable silvicultural interventions (< 90%); therefore, enough wood resource was available across all forest types.

The way the model defines the harvest does have a significant effect on age classes and thus on simulation results (CBM defines the harvest as the amount of carbon targeted, i.e., having a “climate reporting”-oriented approach, whereas EFISCEN defines the harvest in terms of volume, having therefore a “forestry-centred” approach).

### Age-class distribution

Both models showed an increase in forest age over time, whereby in CBM the ageing was much stronger and had less area shift in the regeneration class. Such a dynamic highlights the models’ internalized concept of allocation of harvest demand to forest-management practices, and especially how stand replacements, i.e., final fellings, are distributed according the availability of standing biomass by each of the two models (see Fig. 1).

### Allocation of biomass in other compartments of the stands

Of importance in simulations of standing C stocks and CO_2_ removals were the non-merchantable biomass compartments (e.g., branches, bark, roots, foliage). Despite input-harmonization efforts, CBM simulated an annual average of 46–52% more biomass in these compartments compared with EFISCEN (Table 1). Still, the accuracy of biomass proportions may well be questioned in both cases. EFISCEN uses a straightforward approach in which a BEF value (specific to the forest age and type of non-stemwood biomass compartment) is applied directly to the standing volume parameterized by age and species to estimate the biomass of that compartment. CBM requires as input the proportions of the biomass compartments (i.e., stemwood, bark, branches and foliage) estimated as a function of merchantable volume using a simultaneous fit of all biomass compartments. It is noticeable that employing a simultaneous fitting approach ensures that the sum of compartment predictions equals the prediction of total biomass (so giving due consideration of compartments size within the whole architecture of the standing living biomass). However, both models add other compartments using proportion of the merchantable standing stock, so only the total standing biomass estimate is affected by these proportions.

The quality of the data used to fit these models is also essential; therefore, it is important that the users have access to appropriate data (measured based on robust sampling designs). Instead of using country-specific data to estimate the parameters of Boudewyn equations [58], another alternative is to use the CBM’s Canadian library to select the most suitable parameters [17], although this may result in strange correspondence mapping, e.g., coniferous to broadleaved species. Allocation in compartments could not be reasonably calibrated against any measured data from Romania, but we expect each model is self-consistent (proportions of other biomass compartments stayed rather constant in time).

### The sink in standing living biomass

Overall, there was an enhanced, mutually related model effect on CO_2_ fluxes for the living biomass pool. For example, the annual sinks showed a 15% difference assuming averages during 2010 to 2060, i.e., 17.5 million tCO_2_ in EFISCEN versus 19.3 million tCO_2_ in CBM. Despite different but equally justifiable procedures, there is a cumulative effect when the small, apparently insignificant differences or percentages are applied to the relatively lower carbon stocks in EFISCEN versus the relatively higher carbon stocks in CBM. Such a combined arithmetical effect applies to NAI (e.g., 5% difference), the harvest level achieved (e.g., 2% underachievement of the harvest target by CBM, which means approximately 0.2–0.5 Mt CO_2_ yr^−1^), shares of other biomass compartments and changes in the contribution of forest types with different wood density in the total standing stock (e.g., impact ranges between 1.2 and 2.5 Mt CO_2_ yr^−1^). Apparently, this is in the range of uncertainty of the forest-sink estimates (usually around 20%) [59].

### Mortality and dead wood carbon stock

These two parameters were used to validate the loss from living biomass against the NFI measured data. With regard to carbon stocks in dead wood pools in the initial year, Yasso07 relies on an equilibrium approach (steady state amount), while CBM performs a semi-equilibrium procedure (iteration of saturation levels through repeated disturbances and taking into effect the latest major silvicultural intervention). Default decomposition parametrization of the models was used, with the exception of mortality rate, which was harmonized between the two models. Despite an estimated larger carbon stock in living biomass in CBM (a larger amount of matter transferred to DOM expected given the higher turnover values), the initialization of dead wood pools was lower likely because of a faster decomposition rate in CBM. Uncertainty of C stock in dead wood is expected to increase substantially from standing to lying dead wood, as the NFI only provides volume information, e.g., no decomposition or density information. Obviously, using the same wood density of live tree species would result in overestimation of C stock.

Apparently, both models show an initialization issue with regard to mortality and standing dead wood. Mortality seems higher before the initialization than in simulations because of missing forestry operations and salvage of trees otherwise transferred to dead wood. This seems contrary to the fact that both dead wood stock and merchantable standing stock increased between the NFI-1 and the NFI-2.

### Forest-management practices

The forest-management practices as sampled by the NFI were also simplified for the purpose of simulations. We used a business-as-usual interventions intensity and a regeneration delay after final cut (see Additional file 1: Appendix S2), capturing the patterns of forest types throughout age classes. The two models have a very similar solution to specify silvicultural practices; therefore, these were well harmonized. Nevertheless, our study excluded three types of potential changes that might have influenced the projections: (i) natural disturbances, (ii) wide application of shelter systems and (iii) change of forest composition. The most significant natural disturbance was the windthrow (a stand-replacing disturbance). Given the high rate of salvage logging following windthrow, the impact on age class structure is expected to be low. Therefore, the resulting living biomass pool following windthrow may not be significantly different from typical harvesting. However, we expect significant differences for dead organic matter input, but this is beyond the scope of our study.

The current versions of the models are not spatially explicit, so it is not possible to apply natural disturbances in places where they most likely occur (e.g., in forests affected by wrong past management decisions or forests prone to insect outbreaks). Instead, they are applied randomly across largely defined criteria (forest types and age classes). Furthermore, for comparability between the two models, no shelter systems (which is largely applied in Romanian forestry) and no changes in forest composition were applied (although available from the NFI), as they cannot be easily simulated by either CBM or EFISCEN.

### Specification of deforestation

The two models apply different solutions that may result in either negligible or significant impact on projections depending on the magnitude of deforestation. CBM applies user-defined criteria for deforestation and accounts explicitly for losses in all carbon pools during deforestation, following the IPCC guidance for national GHG inventories [19]. EFISCEN assumes that deforestation takes place after a final felling, so the impact on forest indicators may be significant if there is a different magnitude of deforested area. Nevertheless, this analysis excludes the carbon loss by deforestation in both models. The deforestation rate applied in this simulation seems to have a negligible impact on Romanian forests, as demonstrated by both models.

### Further research, data needs and improvements

The availability of NFI data as regionally aggregated instead of plot-level might have affected the simulation effort. Thus, data and related metadata should be made available freely and openly to the scientific community to ensure good governance of forest and forestry information. Repeated measurements as well as improved modelling tools are further required to allow assimilation of most recent data in the modelling exercises [60].

Both models require pre-processing, and despite they are both open source, the pre-processing may limit the full reproducibility, i.e., if different models are used to fit the yield and increment. For this reason, more transparency is needed when models are improved or used.

A harvest-optimization tool is needed for CBM, which requires explicit harvest demand according to forest types for the simulated period. While this is a straightforward task for non-stand–replacing disturbances based on intensity of thinning interventions, it is a difficult decision for old stands, where forestry principles may result in various responses on forest dynamics: normalization/optimization of forest age structure (e.g., either on standing volume or area), or optimization of transfers to older protected forests (e.g., proportions of area excluded from wood production).

Improvements are already in progress for both models. The new EFISCEN-Space will be based on a modelling approach running on each NFI plot and its individual trees, accounting for information (such as tree densities) and individual tree data (such as diameter and height and running on climate sensitive growth functions). These NFI plot data will allow for better representation of mixed forests, uneven-aged forests, actual forest management and site-specific growth conditions, thereby making a climate-sensitive modelling approach possible. Refining the representation of climate-change impacts is the subject of ongoing research on both models. CBM moves to open-source versions while it strives to implement geospatial simulations [61, 62].

## Conclusions

This model comparison provided valuable insights on both the modelling algorithms and the quality of the input data. In our judgement, both models performed reasonably, providing internally consistent results. While the parallel running on harmonized inputs of these two models provided meaningful insights on related initialization challenges and calibration needs, it also highlights the weakness in data and/or data availability and processing. Despite an as good as possible harmonization of input data, still a 10% difference in sink was found. Therefore, we underline the importance of the input-data quality, the need for further data measurements and model improvement, while the preference for one model or the other should be based on the availability and suitability of the required data.

## Methods

### Input data from the Romanian National Forest Inventory

Data representing the state of the FAWS over 2008–2012, with 2010 as the mid-year of the first Romanian NFI cycle (NFI-1), was used as input into the models. We assumed one general site-class index for the forest growth conditions. The FAWS covers 6.07 million ha, representing about 88% of the total forest national forest area (6.90 million ha). The remaining 12% is either protected, not accessible, not managed or otherwise not available for wood supply. FAWS was stratified by spatial intersection of the seven NUTS-2 (basic regions for the application of regional policies), five climatic units, 10 forest types, two ownership systems (public, private) and two general management strategy categories (high forest or coppice), assumed for 2010. For details, see Additional file 1: Appendix S1. This appendix also indicates how raw data were treated in order to make them compatible to model requirements. Additional file 1: Appendix S2 provides other parameter values, their sources and, when applicable, how they were calculated. Our scenario—a no-natural disturbance, no climate change, but with business-as-usual harvesting level and management scenario—was implemented. For the business-as-usual scenario, present management regimes were implemented (Additional file 1: Appendix S2), using a stable harvest removal level of 23 million m^3^ yr^−1^, at the historical rate of practices across all forest types. Additional file 1: Appendix S3 provides information on the allocation of biomass to other compartments of the standing living biomass. Additional file 1: Appendix S4 provides mortality and decay rates.

### Harmonization of inputs related to volume increment and biomass growth

For each stratum, the age class structure available from the NFI-1 allowed the extraction of the values for forest indicators in terms of area, standing-stock volume and increment, stem mortality rate and dead wood stock (Additional file 1: Appendix S1). CBM initializes the carbon stocks by attaching standing volume from input yield curves defined by the user. It simulates biomass growth based on user-defined volume increment curves and equations for allocation in other biomass compartment as a function of standing volume. EFISCEN, instead, starts from the NFI-reported standing volume on age classes. It projects the growth based on increment through a pre-defined standard procedure. Detailed harmonization of input data regarding the status in the initial year, the processing data procedures, the dynamic of the living biomass and implementation of forest-management assumptions for CBM and EFISCEN are presented in Additional file 1: Appendix S2. To facilitate the consistency of the input data and, therefore, the model comparison, the non-merchantable wood fractions of the tree biomass were harmonized for each forest type given the fundamentally different approaches of the two models. Therefore, for CBM, we defined the functions that predict the proportion (of biomass compartments) as a function of standing volume; for EFISCEN, we defined the fixed percentage values per age class of 10 years. The larger share of stem biomass in CBM (Fig. 1, Additional file 1: Appendix S3) was caused by the way the stem was defined. In CBM, the stem includes the stump, whereas this is not the case in EFISCEN. For conversion of wood volume into biomass, we used the available data on Romanian wood density [63] and the proportions of bark and branches out of total tree volume [64]. The proportion of foliage out of the total standing biomass stock was approximated for each species (or genus) based on CBM default parameters (Additional file 1: Appendix S3).

We harmonized the biomass turnovers and some DOM decomposition parameters (Additional file 1: Appendix S4). The mortality rate was calibrated based on data for 2015 as the mid-year of the NFI-2 (second cycle of the Romanian NFI, 2013–2018), through stepwise modification of turnover values to match the estimated mortality. The starting value for the stepwise procedure was the share of annual mortality for the merchantable carbon stock. The rest of the decomposition parameters and the transfers among DOM pools was based on the default parametrization specific to each model. No further harmonization of the DOM was possible given the totally different concepts of decomposition incorporated. To compile the CBM soil module, we distinguished five climatic units by means of multi-annual averaged annual temperature and precipitation [65]. The EFISCEN soil module uses region-specific climate parameters such as degree days (temperature of the growing season) and the drought index (difference between rainfall and evaporation) [66]. Those parameters are based on the historical weather patterns (i.e., from 1979 to 2017) and were extracted from the European Climate Assessment & Dataset [67, 68].

### Output analysis

To achieve a meaningful comparison of the annual CBM results with the five-year time step of EFISCEN, the CBM results were averaged over five years, e.g., 2011–2015, to correspond to the first age class in EFISCEN (i.e., 2015). Part of the results were analysed in terms of volume (volume is the typical output from EFISCEN), others in terms of carbon estimates (typical output of CBM). In CBM, the conversion from carbon stock (or stock change) back to volume was performed for standing stock and dead wood. The following steps were used to convert the CBM’s carbon output (stock or stock change) back to volume (stock or stock change): (i) calculation of output carbon per hectare (for each stratum); (ii) conversion of carbon per hectare to biomass per hectare; (iii) conversion of biomass per hectare to volume using the inverse of the volume-to-biomass exponential function (parameters hosted in Archive Index Database of CBM), and (iv) aggregation of volume data across entire area of the initial strata, as in [69], which is different to using a constant wood-density value.

In the case of dead wood conversion, we used the actual wood-density factor (which was used to convert volume to biomass in the input data) and a carbon fraction of 50%. We ensured that the comparisons were fully consistent (i.e., refer to the same tree biomass compartments).

Finally, our analysis was based on one business-as-usual scenario for forest-management practices and harvest composition and rate as estimated from NFI-1 and NFI-2.

## Supplementary Information

**Additional file 1: Appendix S1.** Overview of data on the state of forest available for wood supply (FAWS) attached to initial year of the simulation 2010, and of the indicators related to forest change between 2010-2015, and of the indicators related to forestchange between 2010–2015. **Appendix S2.** Harmonization of indicators and parameters relevant for standing stock and growth of living biomass, and forest-management assumptions. **Appendix S3.** Harmonization of allocation of biomass to other biomass compartments of the stands. **Appendix S4.** Harmonization of dead organic matter decomposition parameters, and other relevant inputs into the models.

## Data Availability

The datasets used and/or analyzed during the current study are available from the corresponding author on request.

## Abbreviations

BEF: Biomass expansion factors
CBM: Abbreviation from the CBM-CFS3, Carbon Budget Model of the Canadian Forest Sector
DOM: Dead organic matter
GHG: Greenhouse gases
EFISCEN: European Forest Information SCENario Model
FAWS: Forest available for wood supply
IPCC: Intergovernamental Panel on Climate Change
LULUCF: Land use, land-use change and forestry
NAI: Net annual increment
NFI: National Forest Inventory (IFN in Romanian)
NFI-1: The first Romanian National Forest Inventory cycle
NFI-2: The second Romanian National Forest Inventory cycle
NUTS-2: Nomenclature of territorial units of statistics
tC: Tonnes of carbon
tCO_2_: Tonnes of carbon dioxide
UNFCCC: United Nations Framework Convention on Climate Change
