# Supplementary material for: Two large-scale forest scenario modelling approaches for reporting CO2 removal: a comparison for the Romanian forests
Source: Carbon Balance Manag. 2021 Aug 21;16:25. doi: 10.1186/s13021-021-00188-1 (PMC8379742; doi:10.1186/s13021-021-00188-1)
Supplement: Supplementary file 1 — Additional file 1: Appendix S1. Overview of data on the state of forest available for wood supply (FAWS) attached to initial year of the simulation 2010, and of the indicators related to forest change between 2010-2015, and of the indicators related to forestchange between 2010–2015. Appendix S2. Harmonization of indicators and parameters relevant for standing stock and growth of living biomass, and forest-management assumptions. Appendix S3. Harmonization of allocation of biomass to other biomass compartments of the stands. Appendix S4. Harmonization of dead organic matter decomposition parameters, and other relevant inputs into the models. [file 13021_2021_188_MOESM1_ESM.docx]

**Two large-scale forest scenario modelling approaches for reporting CO_2_ removal: a comparison for the Romanian forests**

Authors: Viorel N.B. Blujdea^1*^, Richard Sikkema^2,3^, Ioan Dutca^1,4^, Gert-Jan Nabuurs^2^

^1^Faculty of Silviculture and Forest Engineering, Transilvania University of Brasov, Șirul Ludwig van Beethoven 1, Brașov 500123, Romania

^2^Department of Forest Ecology and Forest Management (FEM), Wageningen University and Research, Droevendaalsesteeg 3a, 6708 PH Wageningen, the Netherlands

^3^Department of Biological Sciences, University of Limerick, Limerick V94 T9PX, Ireland

^4^Department of Sustainability, Buckinghamshire New University, Queen Alexandra Rd, High Wycombe HP11 2JZ, United Kingdom

*Correspondence: viorel.blujdea@unitbv.ro

** Present address: Department of Biological Sciences, University of Limerick, Limerick V94 T9PX, Ireland

**Additional file 1: Appendix S1. Overview of data on the state of forest available for wood supply (FAWS) attached to initial year of the simulation 2010, and of the indicators related to forest change between 2010-2015.** Data were nationally aggregated or presented as area weighted averages, with sampling error expressed as percentage for 95% confidence interval of the mean, as determined by the Romanian National Forest Inventory (NFI).

| **Forest state indicator** | **Values and description of the inputs** | **Particularities on the data used** |
| --- | --- | --- |
| Forest types and their distribution on areas | The first Romanian National Forest Inventory cycle (NFI-1) estimated FAWS of 6,072,260 ha ± 2% [1], which was stratified in 10 forest types. Three of them were grouped as coniferous: *Abies alba* (10,245 ha), other coniferous (318,365 ha) and *Picea abies* (pure stands with > 90% spruce) (674,483 ha). Four were grouped as broadleaved forests: *Fagus sylvatica* (914,359 ha), other broadleaved (2,303,052 ha), *Quercus robur* (505,508 ha) and *Robinia pseudoacacia* (123,069 ha). Finally, the three remaining ones were grouped as mixed forests: mixed coniferous–broadleaved species (527,284 ha), predominantly broadleaved (> 70% broadleaves species) (330,923 ha) and predominantly coniferous (> 70% coniferous species) (364,980 ha). | FAWS represents 87% of total area forest area estimated by NFI-1. Forest state indicators, e.g., areas, standing volume and net annual increment [NAI], were available on age class of 10 years (e.g., age class 2 includes stands from 10 to 19 years old, etc.). Data were available as spatial intersection of administrative regions (NUTS-2), ownership (public, private), management strategy (high forest or coppice) and five climatic units (which are only relevant for decomposition). |
| Merchantable stock volume and its distribution on forest types | Merchantable stock is defined as the part of the standing growing stock of the forest stands, considered as actually or potentially commercial under current market conditions, measured above a minimum of 5.6 cm diameter at breast height. Includes: all potentially commercial (merchantable) species for domestic markets. The total of 1,502,446,228 m^3^ (247.43 m^3^ ha^–1^) as merchantable stock computed from 2,221,593,469 m^3^ (± 1.8%) for the whole tree volume estimated by NFI-1 (www.roifn.ro). Other broadleaved forests represent 38% of the total standing stock, followed by *F. sylvatica* forests (15%) and *P. abies* forests (11%), roughly proportional to areas attached to each forest types. | NFI provided the volumes corresponding to total aboveground stemwood volume, available as the average values on age classes of 10 years and the 10 forest types defined by seven NUTS-2 regions. |
| Average NAI of the standing stock | Age-class area-weighted net increment of the standing stock (over-bark, FAWS) is 6.86 m^3^ ha^–1^ yr^–1^. The increment varied from 3.4 m^3^ ha^–1^ yr^–1^ for *R. pseudoacacia* to 9.8 m^3^ ha^–1^ yr^–1^ for *A. alba*. | The NFI provided the NAI of the volume corresponding to total aboveground woody biomass (over-bark), i.e., 8,46 m^3^ ha^–1^ yr^–1^, for the entire forest area. The dataset was available as the average values on age classes of 10 years and the 10 forest types defined by seven NUTS-2 regions. Mortality was taken into account in the NFI available data. |
| Annual average felling for 2010–2015 | According to the NFI, the total above-ground volume of harvest was 28.2 million m^3^ (Mm^3^) yr^–1^ ± 10%, which corresponds to 23.1 Mm^3^ yr^–1^ of merchantable wood (e.g., roundwood removals) and to 6.22 million tC (tonnes of carbon). Specifically, 9.3 Mm^3^ (40.3% of total merchantable harvest) or 2.57 MtC (41.3% of total C amount to be harvested) were identified by the NFI as from final felling, while 13.7 Mm^3^ (59.7%) or 3.65 MtC (58.7%)) were identified as from thinning. Data were available per forest type, per age class, per administrative region and per ownership type, as follows: *F. sylvatica* (25%), other broadleaved (19%), *P. abies* (15%), coniferous–broadleaved (11%), predominantly broadleaved (7%), predominantly coniferous (7%), *Quercus sp.* (9%) and other coniferous (4%), *R. pseudoacacia* (2%) and *A. alba* (1%). Values may contain a negligible share of dead trees, as salvage logging, and include the volume removed by deforestation. Available harvest data from the NFI represented a preliminary estimate. | Conversion of NFI estimated standing volume to removed roundwood was done according to wood-exploitation criteria defined in Romanian technical norms (average of 6% for coniferous and 8% for broadleaved species including stumps. Some 10% through bark.). The volume resulting from natural disturbances was included as part of harvest applied, either as thinning or final cut as extracted from the NFI. |
| Annual stem mortality rate | According to NFI-1–NFI-2 (the second Romanian National Forest Inventory cycle), the average mortality was estimated as 0.96 m^3^ ha^–1^ yr^–1^ ± 4.6%. | Mortality represents some 14% of the NAI. |
| Standing dead wood stock for mid-year of NFI-1 (2010) | According to NFI-1, the standing stock of dead wood volume was 53,5 million m^3^ ± 6%, i.e., 8.8 m^3^ ha^–1^. In addition, a lying stock of 68.2 million m^3^ ± 5% is given by the NFI. | The threshold diameter for standing dead wood was 5.6 cm. |

**Additional file 1: Appendix S2. Harmonization of indicators and parameters** **relevant for standing stock and growth of living biomass, and forest-management assumptions for CBM and EFISCEN.** *Note* regarding the harmonization of input data: ^a^ identical inputs in both models; ^b^ harmonized inputs for which the methods used and assumptions taken toward maximizing consistency are detailed in this table.

| **Indicator/parameter** | **CBM** | **EFISCEN** |
| --- | --- | --- |
| **Standing stock and growth** | | |
| Forest area on age/age class in the year of the start of simulation (2010)^a^ | Initialization assumes a uniform distribution of area within the age class (i.e., equal area attached to one-year age step within age class). Distribution is internally achieved by the model. | The state of the forest for each forest type is depicted as an area distribution over the age class and equal volume classes (a matrix model). Initial input matrices require pre-processing by a specific tool (P-EFSOS). |
| Standing volume on age/age class in the year of the start of simulation (2010)^b^ | Such curves are required for the initialization of the standing volume and dead organic matter (DOM) pools in the initial year of the simulation. Standing volume (under-bark) was attached from user-defined yield curves. Age-class dependent, under-bark standing stock volume information was built from NFI data. NFI volume was available for the total woody above-ground part of the trees, so data were converted to standing volume by exclusion of branches and bark percentages in total tree volume (from ref. [2]). Yield curves were obtained by fitting data by Chapman–Richards models [3,4] using the Robustbase package [5] in R [6]. | The actual values of standing merchantable volume (over-bark) are extracted as exact values from the NFI-1. Eventual gaps were filled in by values corresponding to previous age class. Within one age class, the inventory volume is split into a maximum of 10 volume classes to represent the natural variation and allowing the thinning effect to be simulated.  NFI data were converted to standing volume by exclusion of branches for EFISCEN (based on bark and branches percentages in total woody above-ground volume from ref. [2]). |
| Net annual increment in volume of the standing stock for the 10 forest types selected^b^ | Age-class dependent curves of merchantable volume increment (under-bark) were built from NFI data. Such data were fitted by Chapman–Richards models using the Robustbase package [5] in R [6]. Such curves are required to simulate the growth dynamic of the C stock in standing stock. | Growth dynamics are incorporated as five-year NAI as a percentage of the growing stock (over-bark). The coefficients for the growth functions are derived by processing NFI data with the user-selected pre-processing tool. To consider a regrowth after thinning, an extra boost (gamma) default factor is set to 0.4, which means that 40% of the thinned area is moving up one extra volume class, while 60% remains in the same volume class. |
| Merchantable volume to biomass conversion for the 10 forest types selected^a^ | Conversion of merchantable volume to stemwood biomass was done for each of the 10 forest types through fitting an exponential model to merchantable volume and biomass derived by using wood density (according to data from ref. [7]), bark and branches [2] and adding 3% of merchantable volume to account for aboveground stump [8]. For mixed forests, a counterfactual density was estimated for relevant forest types based on the participation of tree species in the forest type based on NFI data. Changes in CBM’s Archive Index Database (AIDB) are according to ref. [9]. | Conversion based on wood density values available on each forest type, |
| Procedures for allocation of biomass to other biomass compartments for the 10 forest types selected^b^ | Biomass of bark, branches and foliage was derived as relative to stemwood biomass following expansion functions of ref. [10]. Parameters for Boudewyn equations were derived through minimization of RMSE by a simultaneous fit of all biomass components to merchantable volume using a Robustbase package in R. Changes in CBM’s AIDB were done according to ref. [9]. CBM default assumptions were used for C stocks in fine and coarse roots [11]. | Age-class–dependent biomass expansion factors for above ground and below ground were estimated for each forest type as the average value on of all data available within an age class. Root to shoot was assumed constant value of 1.18 of total above-ground biomass. |
| **Forest management descriptors** | | |
| Thinning intensity^a^ | Generally, the intensity of silvicultural interventions was set to 15% of the standing volume for early ages (< 40 years), 18% for stands aged 40–80 years and 5% for older stands, as averages of the data extracted from NFI-1–NFI-2. Management-regime prescriptions consisted of defining the minimum and maximum ages for thinning per forest types and, at minimum, an “expert guess” of 5 years since last intervention (no NFI data could be retrieved because of only two cycles of measurements). Thinning events do not modify the age of the stands. | |
| Final cuts characteristics^a^ | Minimum age for the application of final cut was between 25 years for *Robinia sp*. forests and 110 years for *Quercus sp*. forests, assuming that a cut occurs as a single intervention. The assumption was that the felling starts immediately after the end of age range when thinnings apply. In CBM, it was assumed that 97% of standing biomass is transferred to wood products. Final cut events allow reset of the age of the stands to 0 two years after the cut. | |
| Spatial and temporal allocation of thinning and final cut interventions^b^ | Age ranges are defined for each silvicultural intervention for each forest type (and other criteria, such as ownership). An additional rule implemented was that the oldest available stands would be prioritized for harvesting. | EFISCEN distributes the harvest over forest types depending on the available volumes for the predetermined age classes for thinning and felling. If the thinning specifications are too tight, the required volumes will not be reached. As a result, in EFISCEN, the proportion of the harvest that is coniferous decreased until 2060, and there was a corresponding increase in the proportion of the harvest that is broadleaved. |
| Harvest demand^b^ | Defined in terms of a constant amount of C representing the over-bark volume of wood removals, targeted annually for the simulated 50 years. The same wood density was used as for corresponding forest types. The target has to be defined through a combination of constraints as a minimum: forest types, intervention types and age intervals (i.e., detailed allocation of harvest). Volume from deforestation is included in the total amount, but can be differentiated. | Defined in terms of volume of merchantable wood to be harvested at intervals of 5 years, i.e., 116 million m^3^ harvest/5 yr. Total harvest according to intervention types is defined, with the model internally able to distribute the harvest across forest types (i.e., free allocation of harvest), and includes the volume from deforestation. |

**Additional file 1: Appendix S3. Harmonization of** **allocation of biomass** **in other compartments of the stands**

The models demonstrate fundamentally different approaches on allocation of biomass to other biomass, generally non-merchantable, compartments: in CBM, a standing-volume–dependent dynamic input; in EFISCEN, fixed percentage values per age class of 10 years. Specifically, to comply with CBM philosophy on data input, stemwood biomass was complemented with an additional 3% of merchantable stock to account for the above-ground part of the stump. The values of the four biomass sub-pools (stemwood, bark, branches, foliage) on age class were simultaneously fit as function of the merchantable volume by a model mimicking the Boudewyn approach [10]). Average values across all forest types are shown in Fig. 1. However, a specific consistency demand is implicitly implemented through the simultaneous fit of all biomass compartments; failing to do so regularly results in failing runs or unrealistic outputs.


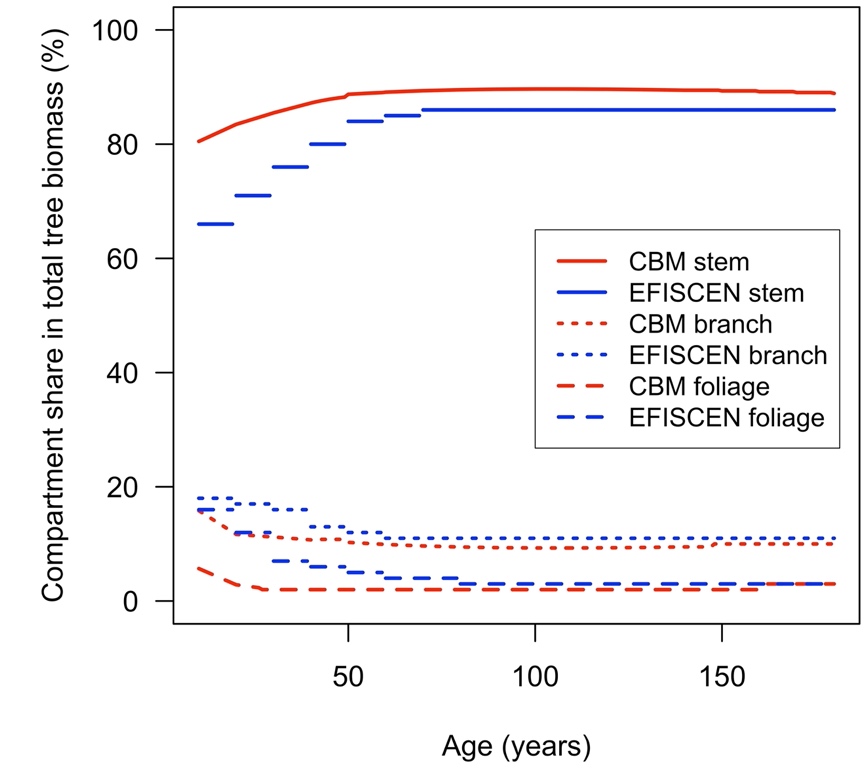


**Fig. 1** Share of biomass components in the total aboveground biomass used as dynamic input in CBM (red) and as horizontal line corresponding to constant values on age-class input in EFISCEN (blue). Data represent the average values across all forest types

**Additional file 1: Appendix S4. Harmonization of dead organic matter decomposition parameters, and other relevant soil inputs into the models.** For ^a^,^b^ marks, see the *Note* as for Appendix B.

| **Indicator/parameter** | **CBM-CFS3** | **EFISCEN** |
| --- | --- | --- |
| Annual stem mortality rate over NFI-1–NFI-2^a^ | Annual mortality rate is 0.30% yr^–1^ for the standing merchantable stock, thus excluding branches and roots. They are only applied to areas that are not subject to any silvicultural interventions. | A mortality rate parameterized per age class and species, i.e., annual mortality rate is 1.491% over 5 years (assuming a cumulative effect of 0.30% mortality per annum), only applied to forest areas not thinned in the previous time step. |
| Annual turnover rates for other biomass compartments^a^ (i.e., transfers from living biomass compartments to DOM) | The model’s default parametrization with regard to decomposition of any pool. Turnover rates are defined for five bio-compartments: branches – 2.7% softwood and 2.5% hardwood; coarse roots (stumps) – 2%; fine roots – 64.1%; and foliage – 25% for softwood and 95% for hardwood species (based on a combination between CBM and EFISCEN default parametrization). | |
| Herbaceous vegetation, undergrowth, lichens | Negligible in terms of carbon stock, but relevant in terms of litter input to the soil. However, for consistency, this is omitted in both CBM and EFISCEN. | |
| Annual standing dead wood fall (decay) rate, i.e., transfer of standing dead wood to litter pool (in % standing dead wood yr^–1^)^a^ | 8.80% yr^–1^. | 36.91% per 5 years (assuming a cumulative effect of 8.80% per year as CBM). |
| Regeneration (i.e., post-harvesting period when no biomass growth was applied) ^b^ | Two years delay from the final cut. | Regeneration is modelled via an average “young forest coefficient”: e.g., 0.75 means that 75% of the forest is regenerated in the first time step attaining first volume class, while 25% remains bare land. About 94% is regenerated over two years. |
| Forests’ composition dynamics^a^ | It is assumed that the initial distribution of forest types stayed the same during simulation. i.e., no tree species composition changes as post-disturbance events. This may give a bias in results of both models, as currently with large spruce decline, forest managers tend to gradually change to more mixed species. | |
| Ingrowth and sub-merchantable trees^a^ | Stemwood biomass for non-merchantable and sapling size trees was not included (it was set to nil amounts in AIDB). | Ingrowth was not included. The volume of sub-merchantable trees can be thinned. |
| Deforestation^a^ | Deforested area of 570 ha yr^–1^, i.e., 0.01% of FAWS area, was fully randomly applied (e.g., no constraint on regions, or forest types or age classes). | Defined per time step of 5 years, i.e., 3850 ha per time step. Deforestation can only occur from bare forest land class with implicit assumption that mature standing stock is taken up in regular harvest. |

**References**

1. NFI, 2018. <http://roifn.ro/site/rezultate-ifn-1/>. Last accessed: 22/06/2021.
2. Giurgiu V, Draghiciu D. Modele matematico-auxologice si tabele de productie pentru arborete [The mathematic and auxologic models, and yield tables for forest stands]. Bucharest, Romania: Ceres; 2004.
3. Fekedulegn D, Mac Siurtain MP, Colbert JJ. Parameter estimation of nonlinear growth models in forestry. Silva Fenn. 1999;33:327–36.
4. Pretzsch H. Forest Dynamics, Growth and Yield. From Measurement to Model. Berlin, Heidelbergy: Springer-Verlag; 2009.
5. Maechler M, Rousseeuw P, Croux C, Todorov V, Ruckstuhl A, Salibian-Barrera M, et al. robustbase: Basic Robust Statistics. R package version 0.93-8, 2021 [Internet]. Available from: http://robustbase.r-forge.r-project.org/.
6. R Core Team (2016) R: A Language and Environment for Statistical Computing. R Foundation for Statistical Computing, Vienna, Austria. https://www.R-project.org/
7. Mos V. Caracteristici fizico-mecanimce ale bazei de materii prime lemnoase din Romania [The phisical and mechanical charateristics of wood resources in Romania]. 1st ed. Bucharest, Romania: Institutul National al Lemnului; 1985.
8. Gschwantner, T., Lanz, A., Vidal, C. et al. Comparison of methods used in European National Forest Inventories for the estimation of volume increment: towards harmonisation. Ann For Sci [Internet]. 2016;73:807–821. Available from: https://doi.org/10.1007/s13595-016-0554-5.
9. Pilli R, Kull SJ, Blujdea VNB, Grassi G. The Carbon Budget Model of the Canadian Forest Sector (CBM-CFS3): customization of the Archive Index Database for European Union countries. Ann Sci [Internet]. 2018 [cited 2019 Sep 20];75:71. Available from: http://link.springer.com/10.1007/s13595-018-0743-5.
10. Boudewyn P, Song X, Magnussen S, and Gillis MD. Model-based, volume-to-biomass conversion for forested and vegetated land in Canada. Victoria, BC, Canada: Natural Resources Canada, Canadian Forest Service, Pacific Forestry Centre. Information Report BC-X-411 ISBN 978-0-662-46513-3, 2007. Cat. no.: Fo143-2/411E
11. Li Z, Kurz WA, Apps MJ, Beukema SJ. Belowground biomass dynamics in the Carbon Budget Model of the Canadian Forest Sector: recent improvements and implications for the estimation of NPP and NEP. Can J For Res. 2003;33:126–136.
